# Immunomodulatory response in an experimental model of brain death

**DOI:** 10.1038/s41598-023-36629-9

**Published:** 2023-06-29

**Authors:** Alexandre Chagas Santana, Wellington Andraus, Dan Zimelewicz Oberman, Nícollas Nunes Rabelo, Filipe Miranda Oliveira Silva, Humberto Dellê, Rafael Pepineli, Edvaldo Leal de Moraes, Cristoforo Scavone, Larissa de Sá Lima, Sabrina Degaspari, Sérgio Brasil, Davi Jorge Fontoura Solla, Liliane Moreira Ruiz, Karina Andrighetti de Oliveira-Braga, Natalia Aparecida Nepomuceno, Paulo Manuel Pêgo-Fernandes, Stefan Gunther Tullius, Eberval Gadelha Figueiredo

**Affiliations:** 1 Neurological Surgery Department, University of Sao Paulo School of Medicine, Av. Dr. Enéas Carvalho de Aguiar, 255, 5Th Floor, São Paulo, CEP 05402-000 Brazil; 2 Organ Procurement Organization Department, School of Medicine, University of São Paulo, São Paulo, Brazil; 3 Gastroenterology Department, School of Medicine, University of Sao Paulo, Sao Paulo, Brazil; 4 Department of Neurosurgery, Hospital de Forca Aerea Do Galeão, Rio de Janeiro, Brazil; 5 Medical Science Department, Nove de Julho University, São Paulo, Brazil; 6 Molecular Neuropharmacology Laboratory, Department of Pharmacology, Institute of Biomedical Science, University of Sao Paulo, São Paulo, Brazil; 7 Cardiopneumology Department, School of Medicine, University of Sao Paulo, Sao Paulo, Brazil; 8 Department of Surgery, Division of Transplant Surgery, Brigham and Women’s Hospital, Harvard Medical School, Boston, MA USA; 9 Division of Neurosurgery, School of Medicine, University of São Paulo (FMUSP), Hospital das Clínicas/FMUSP - Rua Dr. Enéas de Carvalho Aguiar, 255, São Paulo, SP 05403-010 Brazil

**Keywords:** Biomarkers, Health care, Medical research

## Abstract

Liver transplantation has come a long way and is now regarded as the gold standard treatment for end-stage liver failure. The great majority of livers utilized in transplantation come from brain-dead donors. A broad inflammatory response characterizes BD, resulting in multiorgan damage. This process is primarily mediated by cytokines, which increase the immunogenicity of the graft. In male Lewis rats, we evaluated the immune response in a BD liver donor and compared it to that of a control group. We studied two groups: Control and BD (rats subjected to BD by increasing intracranial pressure). After the induction of BD, there was an intense rise in blood pressure followed by a fall. There were no significant differences observed between the groups. Blood tissue and hepatic tissue analyzes showed an increase in plasma concentrations of liver enzymes (AST, ALT, LDH and ALP), in addition to pro-inflammatory cytokines and macrophages in liver tissue in animals submitted to BD. The current study found that BD is a multifaceted process that elicits both a systemic immune response and a local inflammatory response in liver tissue. Our findings strongly suggested that the immunogenicity of plasma and liver increased with time following BD.

## Introduction

The treatment for several clinical conditions marked by severe and irreversible functional and morphological disorder of organs and systems made a major progression in the second half of the last century, with the introduction of organ transplantation, one of the great events in the history of medicine^1^.

However, progress in improving this therapy modality is hampered by a number of obstacles, including a paucity of organs for transplantation, the invasive nature of surgical operations, and a long-term reliance on immunosuppressive medicines, which can cause a variety of side effects^2^.

Currently, the vast majority of organs available for transplantation originate from donors who have been diagnosed with brain death (BD), which has been progressively verified in the literature as having a negative impact on the survival of the transplanted graft^3,4^. In fact, the BD has been defined as the complete and irreversible cessation of neurologic function, and increase of intracranial pressure, which leads to progressive damage entire the cerebrum, brain stem and spinal cord. This phenomenon triggers a parasympathetic activity followed by a severe sympathetic response due to catecholamines release (Cushing’s reflex), capable of causing a variety of systemic changes, including hemodynamic instability, hormonal abnormalities, hypothermia, biochemical and metabolic issues, as well as a widespread inflammatory response^5^. Consequently, such changes contribute to the progressive deterioration of organ function, affecting the viability of the graft in the post-transplant period^5^.

Despite advancements in the understanding of the pathophysiological mechanisms of BD, the immunological mechanisms resulting from BD continue to play a key pathogenic role, threatening the efficacy of this therapeutic technique^3,5^. Therefore, it is accepted that BD is considered an important risk factor for organ transplantation.

The purpose of this work is to analyze and characterize the local and systemic participation of immunologic response in an experimental model of BD, though evaluation of TNF-α, IL-1β, IL-6, IL-10, the quantification of macrophages, NF-κB activity in hepatic tissue, liver allogenicity, and, evaluate liver biochemistry parameters by determining plasma levels of AST, ALT, LDH and ALF in patient with and without BD.

## Materials and methods

All methodology applied in the present study was developed in accordance with national and international standards for the care and use of laboratory animals, with approval from the ethics committee in the use of animals (CEUA—nº 131/17) of the School of Medicine of the University of Sao Paulo.

All experiments reported in this manuscript is in accordance with ARRIVE guidelines.

### Experimental model of brain death

#### Animals

The animal model used in the present study consisted of isogenic strains of Lewis rats (LEW–RT1^1^). The study was conducted in its entirety with male rats, in order to avoid genetic variability related to gender. All animals were obtained from a colony of this strain of rats established in the local vivarium of the Liver Transplantation Laboratory of the School of Medicine of the University of São Paulo (FMUSP). The animals were kept at room temperature of 23 ± 1 °C and a 12/12 h light/dark cycle. In the present study, 16 male Lewis rats were studied, weighing between 300 and 400 g, duly assisted with ventilatory and hemodynamic monitoring.

The experimental model of BD used in this study consists of inducing increased intracranial pressure (ICP) with the aid of a Fogarty® catheter. This model robustly mimics the alterations found in patients diagnosed with BD, becoming a fundamental tool for the study of in vivo pathogenesis, as well as for testing and developing new therapeutic strategies aimed at both the organ donor and recipient.

#### Induction of brain death

The animals were initially weighed and then subjected to the BD protocol described by Kolkert et. al^6^. After anesthetic induction using isoflurane (Forane®, Abbott SA, Buenos Aires; 5%) in a closed chamber, the animals were submitted to orotracheal intubation with a number 6 polyethylene catheter. After, they were ventilated using a mechanical microcontrolled lung ventilator for small animals with the following ventilatory parameters: FiO_2_ = 100%, tidal volume: 10 mL/Kg and frequency of 70 cycles per minute. The animals were placed on a surgical platform with controlled heating (37 °C), in the prone position, after asepsis of the cervical region and skull with 2% iodized alcohol. The right carotid artery was cannulated using a Clay Adams PE-10 polyethylene catheter. The catheter was then connected to a pressure transducer (P23XL Viggo-Spectramed Statham, CA, USA) attached to the monitor (DIXTAL, DX 2021, Brazil) to record the Mean Blood Pressure (MBP) during the entire experimental period. The rational use of the carotid cannulation was based on previous studies performed in rats^7,8^.

Then, a motorized drill was used to perforate the skull (trepanation) and intracranial insertion of a Fogarty-4F catheter (Edwards Lifescience LLC, Irvine, CA, USA) was performed. The effective induction of BD occurred through the rapid inflation of the catheter with 500 μL of saline, being confirmed by the following parameters: hypertensive peak, absence of eyelid reflex, bilateral mydriasis and apnea. After the induction of BD, anesthesia was suspended and the animals remained ventilated for a period of 6 h. The control animals, in turn, were also submitted to trephination, without the insertion of a Fogarty-4F catheter (sham-operated).

#### Subsidiary examination: color transcranial ultrasound

Color transcranial ultrasound assessments were performed with the Micromaxx Ultrasound unit (Sonosite, USA) in conjunction with a 6 to 14 MHz linear transducer. After anesthetic induction of the animals, the left internal carotid artery was cannulated for invasive BP monitoring. Systolic BP values were monitored with the aim of remaining ≥ 90 mmHg and, if necessary, infusion of 0.9% saline solution to correct hemodynamic parameters.

Initially, 2-D ultrasound was used to visualize a B-mode cross-sectional image of the skull as well as the animals' brain structures. Color Doppler ultrasound was then used and the extra and intracranial arteries were indicated on the screen by two different colors (blue and red), where the blue color indicates blood flow away from the transducer and the red color indicates flow towards the transducer. The probe was placed above the skull and the transverse scan was performed by moving the probe in delicate back-to-front motions. The image depth was fixed at 2 cm when applying the magnification. Some settings were also standardized for color doppler ultrasound, such as ultrasound frequency at 6.3 MHz, pulse repetition frequency at 4 kHz, in addition to a frame rate of 65 frames/sec. In addition, a heating lamp was used to maintain a stable body temperature to avoid hypothermia.

**Figure 1:**
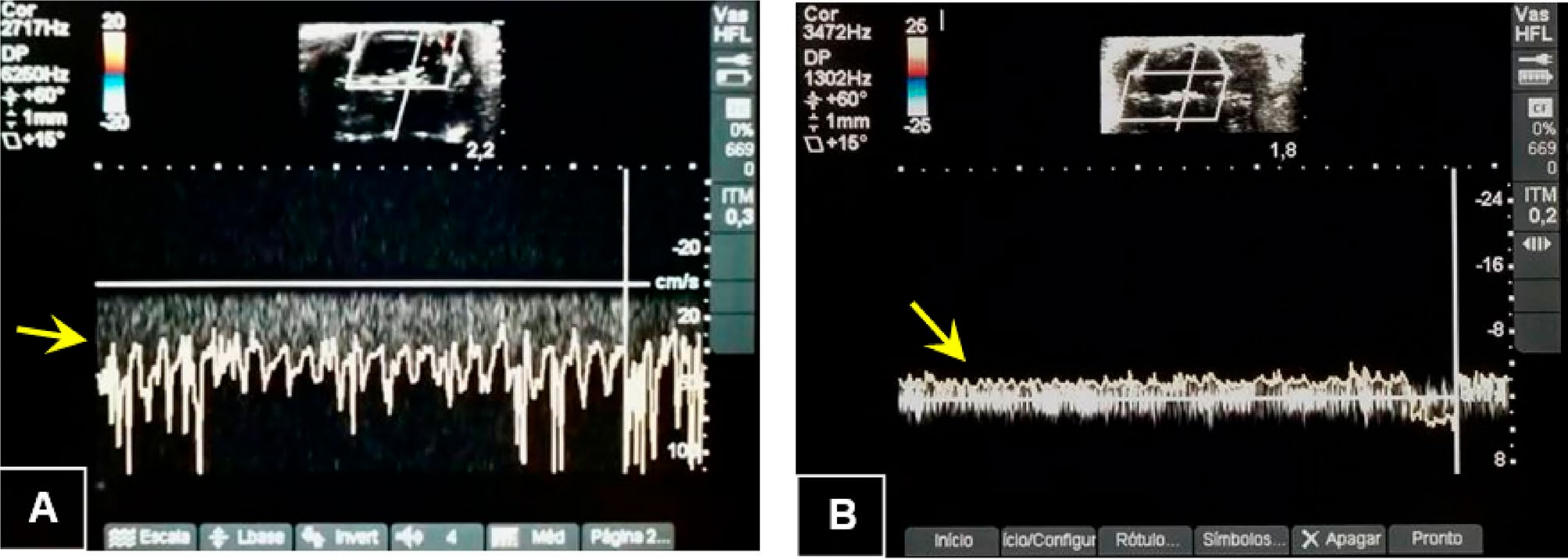
Analysis of cerebral blood flow identified by transcranial color ultrasound. (**A**) Presence of blood flow in the reverse direction (yellow arrow) before brain death. (**B**) Presence of flow compatible with brain circulatory collapse (yellow arrow).

After shaving, the first examination visualized the right internal carotid artery, the right middle cerebral artery, in addition to the basilar artery. Left internal carotid artery and left common carotid artery were not observed/evaluated due to cannulation. The Doppler spectrum was obtained in all the vessels mentioned above (Fig. 1A).

After the Fogarty-4F was inflated, the second examination was performed and monitored until no intracranial vascular signal was obtained, even reducing the pulsed repetition frequency to the lowest achievable value. Systolic peaks below 10 cm/s were recovered in the right internal carotid artery and basilar artery at the level of the skull base, consistent with brain circulatory collapse (Fig. 1B).

### Study design

Six hours after BD, whole blood was collected through the right carotid artery, with the aid of a 3 mL siringe, and immediately transferred to EDTA-treated tube (Fisher Scientific, PA, USA). Plasma was separated by centrifugation (2 °C to 8 °C) at 5000 rpm for 15 min and stored at − 80 °C until assayed. Immediately after blood collection, a median longitudinal laparotomy of approximately 6 cm was performed, followed by opening the abdominal cavity with exposure of the liver. Then, the liver was carefully clamped with the aid of anatomical forceps, and small fragments of liver tissue were carefully removed and stored at − 80 °C for different analyses.

#### Experimental groups

The study was developed with 2 experimental groups as follows:

Control: Animals submitted to trephination without Fogarty catheter insertion (sham-operated) (n = 8).

Brain death: Animals submitted to BD through ICP increase by Fogarty catheter (n = 8).

#### Biochemical determinations

Blood samples were collected from the left carotid artery with the aid of a 3 mL syringe. Plasma concentrations of aspartate aminotransferase (AST) and alanine aminotransferese (ALT) transaminases, in addition to lactate dehydrogenase (LDH) and alkaline phosphatase (ALP) enzymes, were evaluated as indicators of liver abnormalities. The quantification of the results was performed by the optimized ultraviolet method (COBAS MIRA, Roche) according to the protocol of the International Federation of Clinical Chemistry (IFCC). All results were expressed in units per liter (U/L).

### Immunohistochemistry

#### Tissue collection and processing

Liver fragments were carefully dissected and washed with ice-cold saline and fixed in 4% formaldehyde. Then, the fragments were placed in perforated boxes and kept in 10% formalin solution in a phosphate buffer for 24 h until inclusion in paraffin blocks. The inclusion process was performed by an automatic histokinette tissue processor (Jung-Histokinette 2000 Leica, Nussloch, Germany) and lasted approximately 14 h. The process started by dehydrating the tissues in alcohols with progressive concentrations (50% alcohol, 70% alcohol, 96% alcohol (2 baths), and absolute alcohol (2 baths), followed by diaphanization, passing the tissues in a solution of absolute alcohol + xylene and xylol (3 baths), then immersed in molten paraffin at 60 °C. The paraffinized material was included in blocks and remained at room temperature until use.

#### Cutting the tissue fragments

The paraffin blocks were cut on a microtome (Reichert Yung Supercut 2065 Leica, Nussloch, Germany) with disposable razors. The sections, with a thickness between 3 and 4 µm, were adhered to slides previously coated with 2% silane (Sigma Chemical CO, St. Louis, USA), which remained in an oven (Fabbe-Primar, São Paulo, Brazil) at 60 °C for 2 h and then stored at room temperature until use.

#### Deparaffinization

The slides underwent a deparaffinization process, that is, they were kept in xylene for 9 min (Merck, Darmstadt, Germany) 3 times. Then, the slides were dehydrated by bathing in absolute ethanol (2 times) (Merck, Darmstadt, Germany), 96% ethanol (2 times). To finish this process, the slides were immersed in distilled water and processed.

#### Identification of M1 macrophages

M1 macrophages were identified in liver tissue using the mouse anti-monocyte/macrophage monoclonal antibody (anti-ED-1) (Serotec, Raleigh, USA). The LSAB-AP method (Labeled Streptavidin–Biotin Alcaline Phosphatase—Dako, Carpinteria, USA) was used to locate this antibody, and the entire procedure was performed in a humid chamber.

In order to increase antigenic expression, after deparaffinization, the slides were immersed in 10 mM citrate buffer, pH 6.0 and placed in a microwave oven (Sanyo, São Paulo, Brazil) with a power of 2400 watts for a 15 min period. After reaching room temperature, the slides were washed with distilled water to remove the citrate buffer and transferred to TBS (Tris buffered saline), pH 7.6.

To block endogenous biotin, the sections were incubated with avidin D solution (Vector, Burlingame, USA) for 15 min, then washed with TBS for 5 min. Then, the sections were incubated with a biotin solution (Vector, Burlingame, USA) for 15 min and then washed with TBS for 5 min. The sections were then incubated with horse serum (Vector, Burlingame, USA), diluted 70 times in TBS, for 30 min and, after removing excess serum, the sections were incubated with the anti-M1 primary antibody (1:200) during the night period at 4 °C.

The following day, the sections were washed in TBS for 5 min and incubated with the pool of biotinylated anti-mouse, anti-rabbit and anti-goat antibodies (Dako, Carpinteria, USA) for 30 min. After washing with TBS, to complete the reaction, the sections were incubated with the streptavidin–biotin-alkaline phosphatase complex (Dako, Carpinteria, USA) for 30 min and washed again with TBS and destined for development.

For the development, the sections were incubated with a solution containing substrate for the enzyme phosphatase and the fast-red dye (Sigma Chemical Co, Saint Louis, MO, USA), prepared as follows: 1 mg of naphthol phosphate AS-MX (Sigma Chemical Co, Saint Louis, USA) were diluted in 100 µL of dimethylformamide (Merck, Darmstadt, Germany). Then, the solution was diluted in 4.9 mL of 0.1 M Tris buffer (pH 8.2) and 10 μL of 1 M levamisole (Sigma Chemical Co, Saint Louis, USA) were added. This solution, in turn, remained stored at − 20 °C and, at the time of development, it was mixed with 5 mg of the fast-red dye.

The development was performed under microscopy at 200X magnification and the positive cells showed brown staining. The development time was approximately 10 min. Slides were counterstained with Mayer's hemalumbre (Merck, Darmstadt, Germany) for 2 min, washed in distilled water and mounted with glycergel (Merck, Darmstadt, Germany).

#### Analysis of slides

All slides were analyzed so that the researcher was not aware of which group would be analyzed. The analysis of staining for M1 macrophages was performed using the quantitative method, i.e., positive cell counts (stained in brown) were performed throughout the tissue inserted in the slide, under microscopic magnification of × 400 and expressed as cells/mm^2^.

### Gene expression

#### Total RNA extraction

All solutions used for RNA extraction were used with deionized water through the Milli-Q system (Millipore, Milli-Q Element A10 System, Massachusetts, USA) and treated with diethylpyrocarbonate (Sigma, Sto Louis, USA). For each liter of deionized water, 1.0 mL of diethylpyrocarbonate were added and the solution was kept at 60 °C under stirring for approximately 12 h, then autoclaved (121 °C for 20 min). Total RNA from liver tissue was reacted with Trizol (Invitrogen, California, USA), following the protocol suggested by the manufacturer. Each 100 mg of tissue was homogenized with 1 mL of Trizol using a tissue disperser (IKA—Labortechnik Ultra Turrax T25 Janke & Kunkel, Germany). For each mL of homogenate, 200 µL of chloroform (Merck, Darmstadt, Germany) was added. Then, the mixture was homogenized again and kept for 3 min at room temperature.

After incubation, the mixture was centrifuged at 12,000×*g* at 4 °C for 20 min. The upper RNA-containing phase was transferred to a 2 ml microtube containing the same volume of ice-cold isopropanol (Sigma Chemical Co, Saint Louis, USA). The samples were centrifuged at 12,000×*g* for 10 min, the supernatant was discarded, the RNA pellet resuspended in 1 mL of 70% ethanol (Merck, Darmstadt, Germany) and centrifuged again at 12,000×*g* for 10 min. This procedure was repeated and the pellet resuspended with 50 µL of water previously treated with diethylpyrocarbonate.

#### Quantification of total RNA

RNA quantification was performed in a spectrophotometer (NanoDrop, Thermo Fisher Scientific, Marietta, USA) measuring absorbance at wavelengths 260 and 280 nm. The RNA concentration expressed in µg/mL was calculated from the absorbance at 260 nm. The reading of 1 OD corresponds to a pure solution of single-stranded RNA at a concentration of 40 µg/mL. Reading at 280 nm was used to determine protein contamination of samples. The analysis was based on the ratio between the absorbances at 260 and 280 nm, and the acceptable value is 1.7 to 2.0.

#### cDNA synthesis

All reagents used for the complementary DNA (cDNA) synthesis reaction were Promega (Promega, San Luis Obispo, USA). One microliter of oligo dT primer (500 µg/mL) was mixed with 11 µL of a 50 ng/µL RNA solution. The solution was heated at 70 °C for 10 min and cooled on ice for 5 min. Then, 4 µL of [5X] buffer (250 mM Tris–HCl pH 8.3, 375 mM KCL, 15 mM MgCl_2_), 2 µL of 0.1 M DTT, 1 µL of dNTP Mix (10 mM dATP, dGTP, dCTP and dTTP) and 1 µl (200u) of the M-MLV (Moloney Murine Leukemia Virus) reverse transcriptase enzyme. The reaction was carried out at 42 °C for 50 min, followed by a period of 15 min at 70 °C for the inactivation of the enzyme. The cDNA was kept in a freezer at – 20 °C until the real-time PCR reaction was performed. For this step, pairs of primers were made to obtain amplicons with a maximum of 250 bp, as recommended for real-time PCR.

#### Real-time PCR: qPCR

In this protocol, 1 µL of cDNA was added to 7.5 µL of kit mix, 0.6 µL of forward primer (10 µM), 0.6 µL of reverse primer (10 µM), 5.75 µL of deionized water and 0.15 µl of Rox. All reactions were performed in triplicates. The mixture was heated to 50 °C for 10 min and then to 95 °C for 5 min, following 40 cycles of 95 °C for 15 s, 60 °C for 30 s and 72 °C for 30 s. The melting curve was performed at 65 °C with a variation of 1 °C. The equipment used was the StepOnePlus™ Real-Time PCR System (Applied Biosystems, Singapore). Gene expression was determined as the relative expression between the target gene and the β-actin housekeeping gene, calculated by the computer program of the StepOnePlus™ Real-Time PCR System (Applied Biosystems, Singapore), from the cycle thresholds (CT) of the reactions. The sequence of primers used for Real Time PCR and Cycle Treshold used for Real Time PCR assays are show in Supplementary File 1.

#### Multiplex/luminex cytokine analysis

The quantification of cytokines was performed using the multiplex/luminex method with the MILLIPLEX® MAP kit (Millipore Corporation, Billerica, MA, USA). The assay was performed on the liver fragment of the animals. The Rat panel (cytokine/chemokyne—immunoassay), composed of antibodies, was used to quantify the following cytokines: TNF-α, IL-1β, IL-6 and IL-10, MHC Class I, MHC Class II and NFκ-B. Assays were performed according to the manufacturer's protocol.

Initially, for the extraction of total protein, the liver tissue fragment was pulverized in a glass pestle, containing liquid nitrogen and resuspended in 1 mL of protein lysis buffer (RIPA Buffer, Millipore®) together with the protease inhibitor. Then, the samples were left to rest for 30 min at 4 °C. After this period, the samples were centrifuged at 10,000 rpm for 30 min at 4 °C.

The supernatant was then collected and stored at − 80 °C until measurement. The filter plate containing 96 wells was washed with Bioplex Wash Buffer. Then, beads conjugated with anti-cytokine antibodies were added and washed with Bioplex Wash Buffer, and the previously prepared samples were added later.

The samples were incubated for 2 h and then washed with Bioplex Wash Buffer. Then, biotinylated antibody was added to each sample and incubated for 1 h and new washes were performed with Bioplex Wash Buffer. For the analysis of the results, the Bio-Plex Manager Software, version 4.0 (Bio-Rad) was used in the Bioplex Suspension Array System/Luminex (Bio-Rad) system.

#### Electrophoretic mobility delay test for NFκ-B

This assay (EMSA or Gel-Shift) consists of the binding reaction of nuclear extract proteins with a specific nucleotide sequence for the transcription factor, which was previously labeled with 32P by the T4 polynucleotide kinase. When the reaction medium is subjected to polyacrylamide gel electrophoresis, the free probe (oligonucleotide) migrates more than the probe bound to the nuclear factor (lag band).

##### Extraction of nuclear proteins from liver tissue

The method used was described by Rong et al.^9^. Cells were harvested in ice-cold PBS and centrifuged at 2000 g × 5 min × 4 °C and the pellet was resuspended in 400 µl of lysis buffer (10 mM HEPES; 1.5 mM MgCl_2_; 10 mM KCl; 2 µg/mL leupeptin; antipain 2 µg/mL; 0.5 mM PMSF; 0.1 mM EDTA) and incubated on ice for 15 min. 10 µL of 0.5% NP-40 was then added under vigorous agitation, centrifuged at 13,000×*g* for 30 s at 4 °C, and then the supernatant was discarded.

The pellet was resuspended in 20 µL extraction buffer (20 mM HEPES; 1.5 mM MgCl_2_; 300 mM NaCl; 0.25 mM EDTA; 2 µg/mL leupeptin; 2 µg/mL antipain; 0.5 mM PMSF) and incubated for 20 min on ice under agitation, centrifuging the extract at 13,000×*g* for 20 min at 4 °C. The supernatant was collected and the protein concentration determined by storing the samples at − 80 °C.

#### Probe marking

The DNA oligonucleotide containing the sequence (5'-AGT TGA GGG GAC TTT CCC AGG C-3') was labeled with the addition of -32P ATP in a solution containing T4 kinase buffer, T4 kinase and water at concentrations of: 3, 5 pmol oligonucleotide, 1 U/µL T4 kinase, 1 µL α-32P ATP (3 Ci/mmol), 1 µL T4 Kinase Buffer buffer (10x) in 10 µL reaction volume. After incubation at 37 °C for 10 min, excess -32P ATP was removed with Sephadex G-25 resin. Columns (Microspin G-25) were placed in a microcentrifuge tube and centrifuged for 1 min at 3000 rpm. The column was transferred to a new tube by applying the labeled probe to the center of the resin. After centrifugation the eluate was collected, and on the day of the assay the activity of the probe was determined, using approximately 30,000 cpm/µL in the assay.

#### Binding reaction and gel run

Four µL of 5 × binding buffer was added to a tube (5 mM MgCl_2_; 2.5 mM EDTA; 2.5 mM DTT; 300 mM NaCl; 50 mM Tris–HCl pH 7.5; Poly dIdc 0.25 µg/µL and 20% glycerol); nuclear extract in sufficient quantity for 5 µg of protein; excess cold oligo; and H2O q.s.p. to 20 µL of final volume (including addition of labeled probe).

The tube was then incubated for 20 min at room temperature, then the labeled probe (1 µL) was added and incubated again for 30 min at room temperature. The run was visualized with the addition of 1 µL of Bromophenol Blue to the negative control. The entire content of the reaction medium was applied to the 5.5% polyacrylamide gel (acrylamide/bisacrylamide (37.5:1)). For electrophoresis a running buffer consisting of 0.5 × TBE (1 × TBE = 90 mM Tris, 90 mM Boric Acid, 1 mM EDTA) was used. The gel ran for about 2 h at 150–160 V. At the end of the run, the gel was dried and the film was then exposed to the gel in a cassette at − 80 °C.

#### Super-shift tests

The NF-κB delay gel Super-Shift assays were performed in an identical manner to the procedures described above. Specific antibodies were added to the subunits that make up the NF-κB complex, during the incubation process of the nuclear extract with the oligonucleotide, containing the specific sequence of NF-κB. For this, p50, p65 and c-Rel antibodies (Santa Cruz, CA, USA) were used.

#### Statistical analysis

The normality of data distribution was verified using skewness, kurtosis statistics and graphical methods. SPSS statistical software (version 24.0; IBM, Armonk, New York, USA) was used to perform the statistical analysis. Statistical analyzes of the gel-shift results were performed using the Prism statistical program (GraphPad, San Diego, USA). ANOVA was used for pairwise comparisons according to the Newman-Keuls formulation. All results were presented as mean ± standard error. Statistical significance was considered from p < 0.05.

## Results

### Analysis of mean blood pressure (MBP)

**Figure 2:**
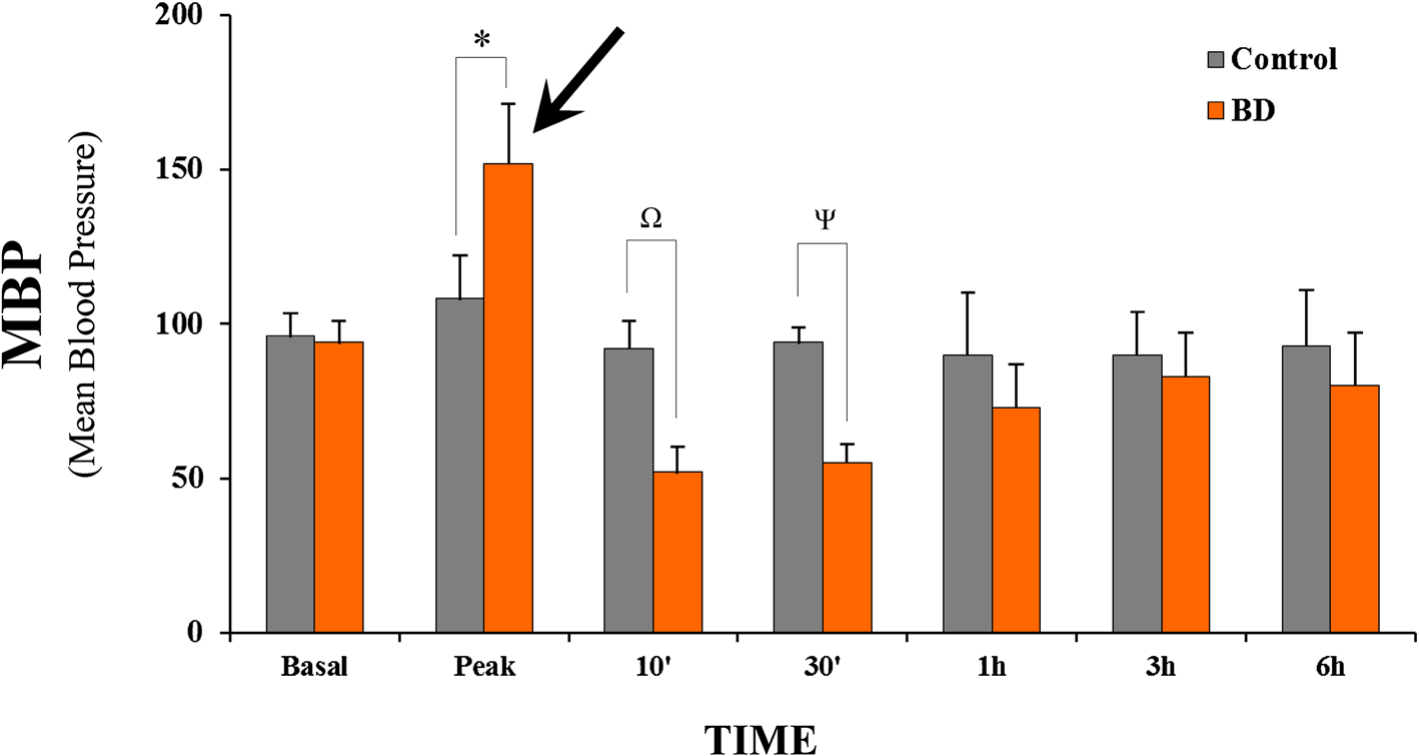
Comparative analyzes of the evolution of MBP between the groups during a period of 6 h after the induction of brain death (*MBP* mean blood pressure).

The mean MBP measured in the BD group prior to inflation of the balloon was 94.0 ± 6.9 mmHg, similar to the controls (96.1 ± 7.5 mmHg). After BD induction, there was an intense rise in blood pressure followed by a fall, indicating intense activity of the sympathetic autonomic response (black arrow). The mean MBP values in the BD group were maintained above 50 mmHg, and control group above 94 mmHg, throughout the follow-up period, without the need for vasoactive drugs or colloids. Although the animals in the control group showed higher MBP in the the different times analysed, no significant differences were observed between the groups 1 h after BD induction. The results are illustrated in Fig. 2.

### Analysis of plasma concentrations of AST, ALT, LDH and ALP

The biochemical analyzes in the plasma of the studied groups revealed that the animals of the BD group had significantly elevated values of AST, ALT, LDH and ALP, when compared to the control groups (Table 1).

**Table 1:** Comparative analysis of liver damage indicator enzymes 6 h after brain death induction.

Parameters (IU/L)	Groups	
	Control	BD
ALT	66.6 ± 9.2	232.7 ± 40.9*
AST	140.1 ± 3.9	349.2 ± 26.5*
LDH	267.1 ± 17.7	411.1 ± 12.4*
ALP	99.2 ± 9.5	135.8 ± 13.2*

*ALT* alanine aminotransferase, *AST* aspartate aminotransferase, *LDH* lactate dehydrogenase, *ALP* alkaline phosphatase.

p < 0.05 vs. Control. ALT: *p = 0.0001; AST: *p = 0.0001; LDH: *p = 0.0001; ALP: *p = 0.0273.

### Immunohistochemistry for M1 Macrophages

**Figure 3:**
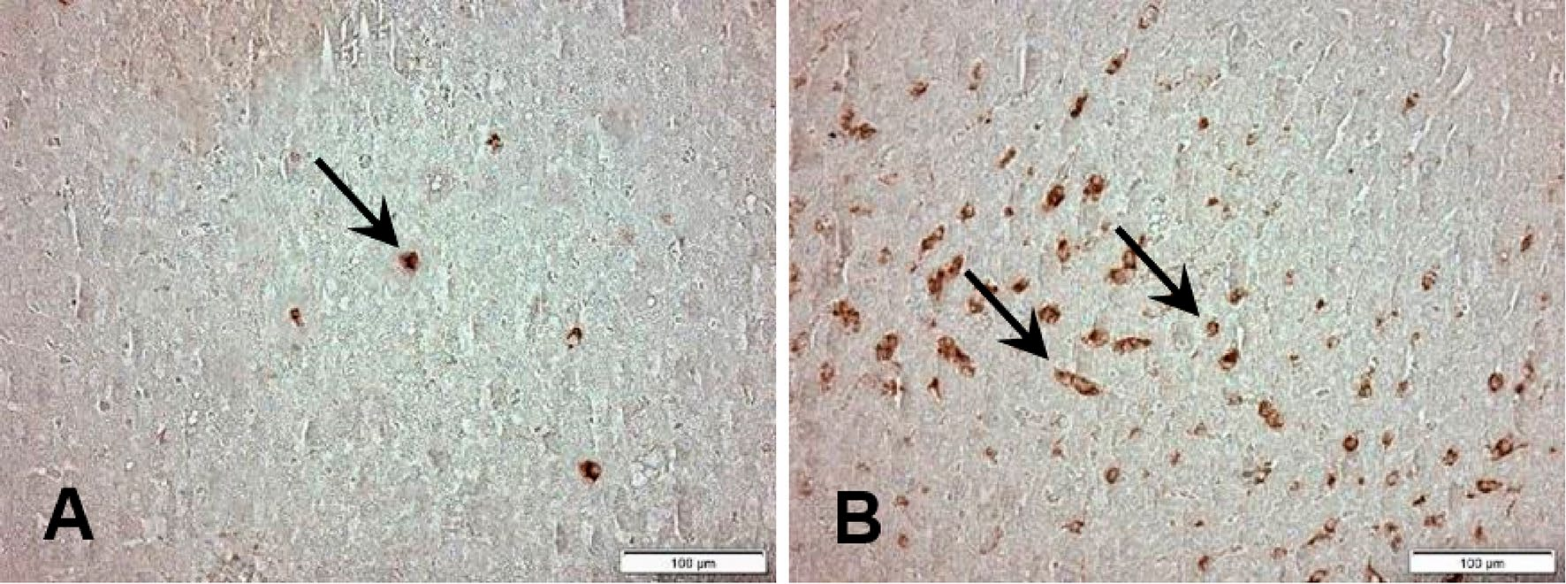
Immunohistochemistry for detection of M1 macrophages (arrows) in liver tissue 6 h after induction of brain death. (**A**) Control; (**B**) Brain death; 400X magnification.

**Figure 4:**
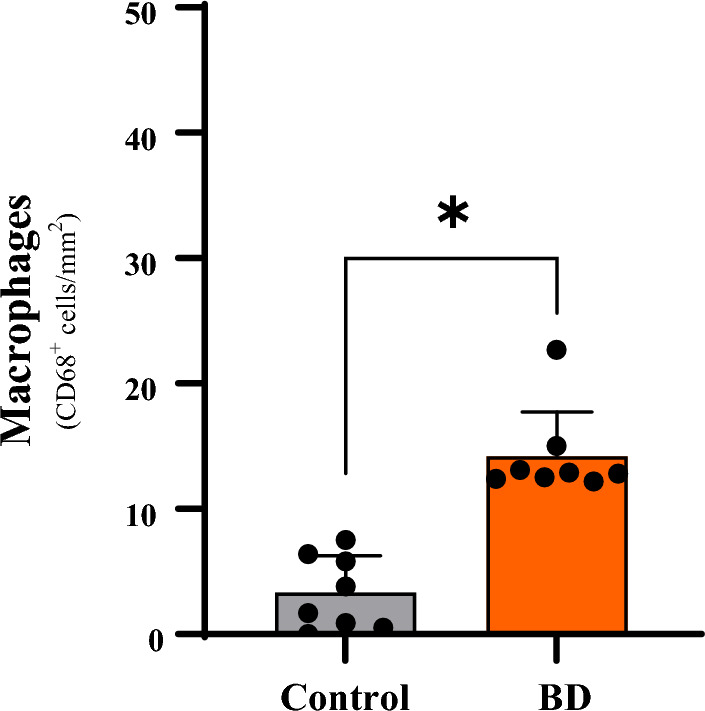
Quantification of M1 macrophage infiltrate 6 h after brain death induction in different experimental groups. *p < 0.05 vs. Control; (*BD* brain death).

In the BD group there was a significant macrophage infiltrate in the liver tissue compared to the control group (14.2 ± 1.3 vs. 3.3 ± 1.0 cells/mm^2^; p < 0.05). Representative microphotographs are illustrated in Figs. 3A,B. The quantification of these macrophages is shown graphically in Fig. 4.

### Analysis of inflammatory mediators in liver tissue

In order to identify the cellular and molecular mechanisms involved in liver tissue during BD, inflammatory mediators were analyzed by real-time PCR and MULTIPLEX/LUMINEX assays. The pro-inflammatory cytokines TNF- α, IL1- β and IL-6 were quantified, in addition to the anti-inflammatory cytokine IL-10.

In general, the animals in the BD group showed significantly increased levels of gene expression and protein concentration of cytokines TNF- α, IL-1 β and IL-6 in relation to the control group, indicating an intense local immunological process.

**Figure 5:**
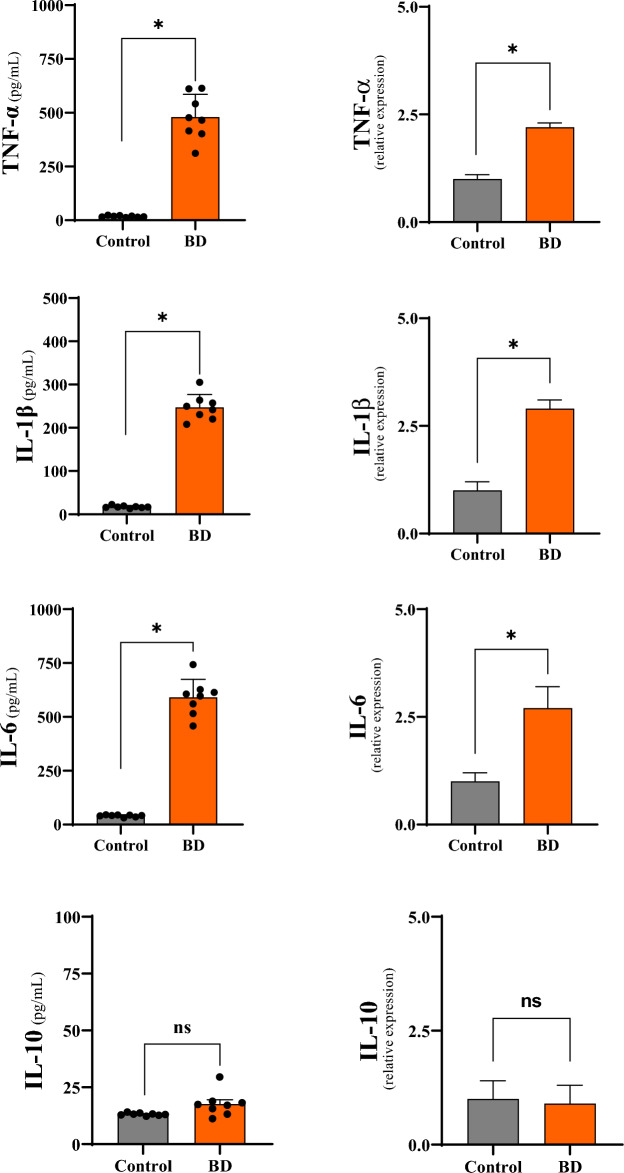
Comparative analyzes of gene expression and concentration of inflammatory meters in liver tissue 6 h after brain death in the different groups. *p < 0.05 vs. Control; #p < 0.05 vs. BD (*BD* brain death).

The analysis of IL-10 expression, in turn, did not show a significant difference between the groups. The results are expressed in Table 2 and graphically illustrated in Fig. 5.

**Table 2:** Comparative analysis of inflammatory mediators in liver tissue 6 h after brain death induction in different groups.

Cytokines	Groups			
	Control		BD	
	mRNA (relative expresión)	Protein (pg/mL)	mRNA (relative expresión)	Protein (pg/mL)
TNF-α	1.0 ± 0.1	17.9 ± 1.9	2.2 ± 0.1*	478.8 ± 109.5*
IL-1β	1.0 ± 0.2	17.3 ± 2.4	2.9 ± 0.2*	247.0 ± 29.8*
IL-6	1.0 ± 0.2	40.8 ± 4.8	2.7 ± 0.5*	590.5 ± 87.4*
IL-10	1.0 ± 0.4	13.2 ± 0.6	0.9 ± 0.4	17.6 ± 2.2

*BD* brain death, *TNF* tumor necrosis factor.

*p < 0.05 vs. Control. mRNA (TNF-α: *p = 0.0001; IL1-β: *p = 0.0001; IL-6: *p = 0.0001; IL-10: *p = 0.6248).

Protein (TNF- α: *p = 0.0013; IL1- β: *p = 0.0001; IL-6: *p = 0.0001; IL-10: p = 0.0812).

### Class I MHC and Class II MHC detection

**Figure 6:**
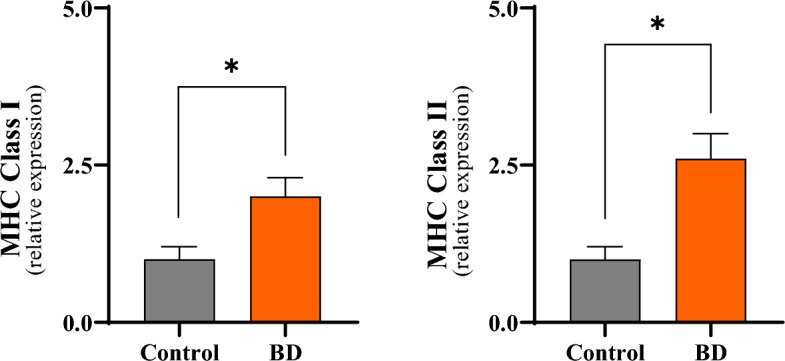
Comparative analyzes of Class I MHC and Class II MHC expression in liver tissue 6 h after brain death in the different experimental groups. *p < 0.05 vs. Control; #p < 0.05 vs. BD (*BD* brain death, *MHC* major histocompatibility complex).

The expression in the liver tissue, determined by real-time PCR, of two important molecules related to immunogenicity in organ transplantation, Class I MHC and Class II MHC is shown in the Fig. 6. The hepatic expression of each of these molecules increased significantly in the BD group compared to the control group, being significantly attenuated. The results are shown in Table 3.

**Table 3:** Expression of Class I MHC and Class II MHC in liver tissue 6 h after brain death induction in the different groups.

mRNA (relative expression)	Groups	
	Control	BD
MHC Class-I	1.0 ± 0.2	2.0 ± 0.3*
MHC Class-II	1.0 ± 0.2	2.6 ± 0.4*

*BD* brain death, *MHC* major histocompatibility complex.

*p < 0.05 vs. Control. MHC Class I: *p = 0.0001; MHC Class II: *p = 0.0001).

### Plasma concentration of inflammatory mediators

In order to evaluate the systemic repercussion of the immune response in this experimental model, the pro-inflammatory cytokines TNF- α, IL1- β and IL-6, in addition to the anti-inflammatory cytokine IL-10, were measured in the plasma of the animals by means of MULTIPLEX/LUMINEX assays. The analysis revealed that the animals of the BD group showed a significant increase in the plasma concentration of TNF- α, IL1- β and IL-6 in relation to the animals of the control group. IL-10 analyzes revealed no significant differences between groups. The results are expressed in Table 4.

**Table 4:** Comparative analysis of inflammatory mediators in plasma 6 h after brain death induction in the different groups. (BD: Brain death).

Parameters (pg/mL)	Groups	
	Control	BD
TNF-α	2.1 ± 0.4	46.8 ± 10.4*
IL-1β	0.8 ± 0.4	27.9 ± 4.0*
IL-6	2966.5 ± 793.7	9310.0 ± 976.8*
IL-10	307.7 ± 70.3	355.3 ± 94.6

*p < 0.05 vs. Control. (TNF-α: *p = 0.0007; IL1-β: *p = 0.0001; IL-6: *p = 0.0002; IL-10: *p = 0.6924).

### Analysis of NF-κB expression and activity

**Figure 7:**
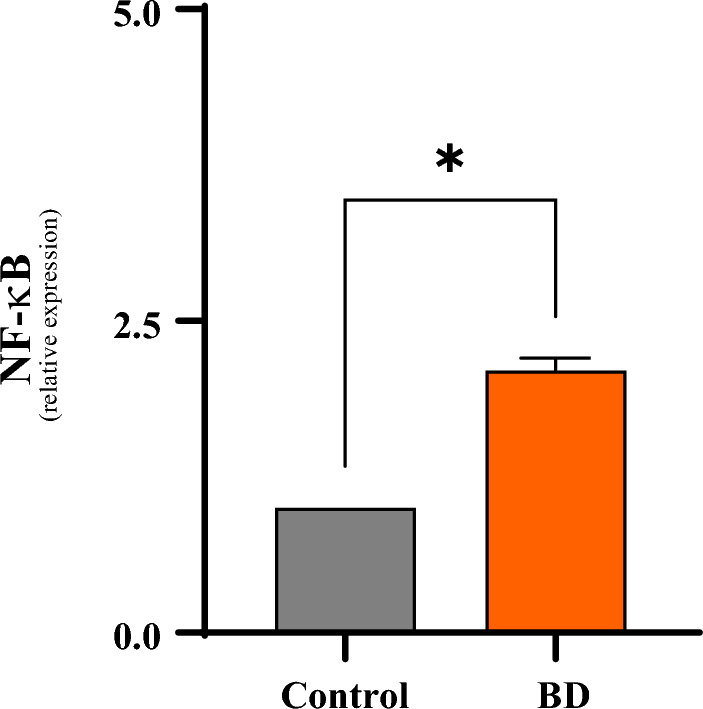
Comparative analyze s of NFB gene expression in liver tissue 6 h after brain death in different groups. *p < 0.05 vs. Control; ^#^p < 0.05 vs. BD. (*BD* brain death).

For a better understanding of the molecular mechanisms involved in BD, an analysis of the expression of the transcription factor NF-κB was performed by the RT-PCR and Gel-Shift methods in liver tissue. NF-κB gene expression was significantly higher in the BD group compared to the control group (1.0 ± 0.1 vs. 2.1 ± 0.1 relative expression; p < 0.05) (Fig. 7).

**Figure 8:**
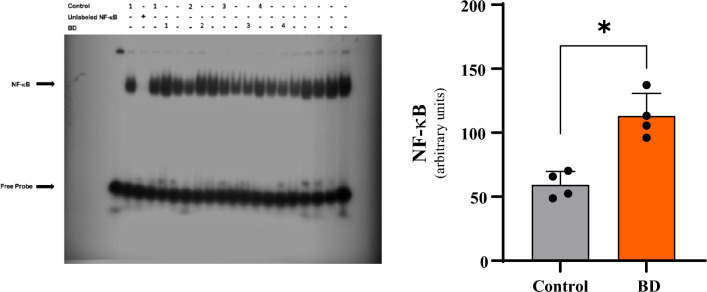
Analysis of the densiometric quantification (arbitrary units) of the NF-αB band in the liver tissue in the different groups. *p < 0.05 vs. Control; ^#^p < 0.05 vs. BD (*BD* brain death).

In parallel, analyzes by the Gel-Shift assay showed that the animals in the Control group had barely visible bands. In contrast, the animals in the BD group showed dense bands, indicating activation of the NF-κB transcription factor. The quantification of these bands by densitometry is shown graphically in Fig. 8, in which the differences pointed out are more clearly visible.

### Analysis of p50, p65 and c-Rel subunits

**Figure 9:**
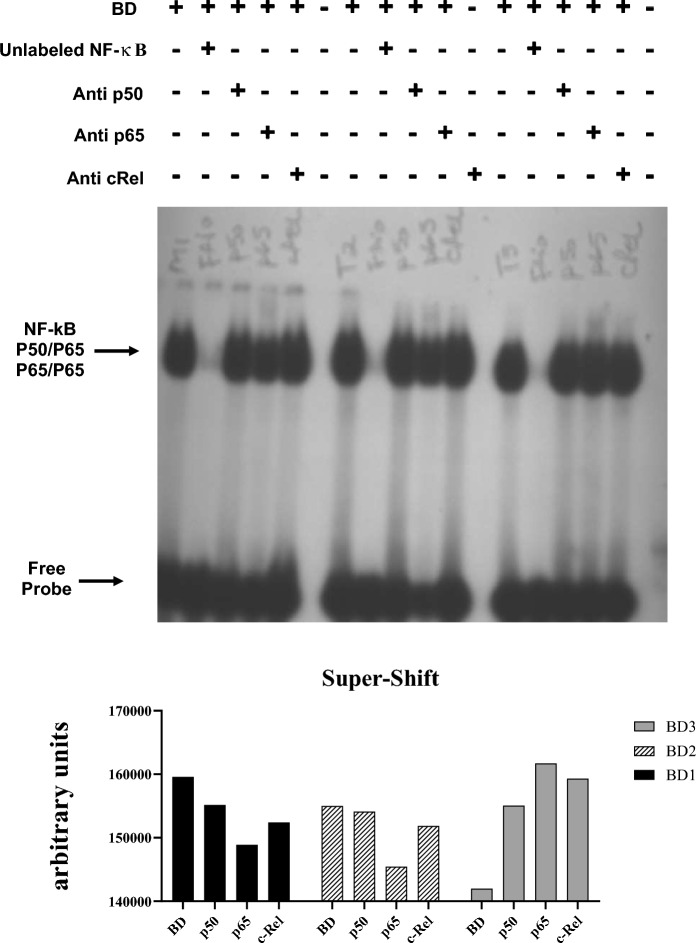
Illustrative image of the Super-Shift assay 6 h after brain death induction (BD group), indicating that the dimers present in liver tissue are composed of the p50, p65 and c-Rel subunits (*BD* brain death).

For the subunits involved in the activation of NF-κB, the Super-Shift assay was performed to identify the composition of the dimers that were present in the nucleus of liver tissue cells. Super-Shift analysis indicated that antibodies against the p50 and p65 subunits induced a partial decrease in the NF-κB complex. On the other hand,the presence of antibodies against the c-Rel subunit did not affect the DNA–protein complexes. Therefore, taken together, these results indicate that p50/p65 heterodimers and p65/p65 homodimers were inserted into the NF-κB/DNA complex. Representative radiographic images from the Super-Shift assay are illustrated in Fig. 9.

## Discussion

Organ donation for transplantation has evolved over the years, especially in light of the development of BD diagnosis^10,11^. BD, in turn, is considered the initial event of a series of changes of a biochemical, biophysical, molecular and immunological nature, responsible for progressive deleterious effects on the structure and function of potentially transplantable somatic organs^12^. Among these effects, cardiovascular abnormalities, hormonal changes, metabolic derangements, as well as immunological events stand out^10,11^.

The focus of treatment for these disorders has mostly been on hemodynamic support and endocrine-metabolic regulation, with little attention paid to immune response maintenance. Several investigations have found that the immune response elicited by BD is closely linked to graft immunogenicity and, as a result, the potential impact of transplanted organ survival^12^. Furthermore, the complicated immunopathological alterations linked with BD in peripheral organs are still poorly characterized^13^.

We used an experimental model of BD to elucidate the complex immunopathogenic mechanisms involved in this condition, which reproduces the main characteristics observed in humans diagnosed with BD and is thus a crucial tool for in vivo studies, as well as tests and analysis of new therapeutic strategies.

Initially, we believed that NFκB would play a key role in controlling the immune response in peripheral organs, and that thus inhibiting its activity might protect liver tissue against immunogenicity. In reality, NFκB regulates genes involved in innate and adaptive immunity, apoptosis, and cell adhesion, among other things, according to clinical and experimental findings^12,14^.

Nonetheless, our findings revealed that the BD experimental model is highly repeatable within a strictly technical methodological sequence. We also did a color transcranial ultrasonography as a follow-up assessment to confirm the diagnosis of BD. The intracranial arteries (internal cerebral artery, right middle cerebral artery, and basilar artery) were examined, and blood flow was interrupted in all three vessels, indicating brain circulatory collapse. Few experimental investigations use supplementary tests for the diagnosis of BD because they require a skilled operator.

Blood pressure monitoring revealed that the animals had a significant increase in MBP shortly after BD induction, as expected. The animals thereafter demonstrated a drop in blood pressure, which remained normal and regulated during the follow-up. An severe unrestrained secondary sympathetic activity (adrenergic storm) of brief duration is known to cause an elevation in blood pressure during the early stages of BD, a fact that implies a secondary and transient connection to brainstem ischemia^15,16^. As a result of the damage to the central vasomotor structures induced by generalized neuronal dysfunction, there is a decrease in catecholamine levels in the blood, resulting in decreased inotropism and chronotropism, low cardiac output, and, as a result, lower blood pressure^16^.

Our blood tissue analyzes showed an increase in plasma concentrations of liver enzymes (AST, ALT, LDH and ALP), in addition to pro-inflammatory cytokines (TNF- α, IL-1 β and IL-6) in animals submitted to BD, corroborating previous studies^12,16^. These findings support the hypothesis that the neurological insult triggers a neuroinflammatory response, resulting in functional impairment of the blood brain barrier, which predisposes a bidirectional flow of immune components between the central nervous system (CNS) and the systemic circulation^17^. CNS-derived cytokines (TNF- α, IL-1 β and IL-6) are free to interact with receptors located on the surface of peripheral tissues, leading to the recruitment of inflammatory cells that induce a local intracellular response.

Endothelial cells have the potential to rapidly synthesis and release cytokines and growth factors in the presence of this inflammatory environment, which are effective modulators not only in the immunological context, but also in the regulation of hemostasis and thrombosis occurrences^18^. In fact, the endothelium interacts with circulating platelets, which form clumps with leukocytes, obstructing the microcirculation and causing direct ischemia damage^18^.

Pro-inflammatory factors also have relevant effects on liver cells, inducing the synthesis of chemical mediators (CCL2/MCP-1 and CXCL1), class II MHC expression, apoptosis, among others^5,19^. The local production of these molecules is important because, in addition to increasing the infiltration/expansion of macrophages in the interstitium, they cause cell damage and decline in liver and vascular function^19^.

As a result, substances produced by liver cells might be utilised by the cells themselves (autocrine effect) or by cells nearby (paracrine effect), continuing the immune response and functional problems. That is, there was damage to the liver tissue as a result of the systemic immunological phenomena, which resulted in an increase in enzymes in the circulation.

In the present study, we also observed accumulation of macrophages in the liver, identified by immunohistochemistry. This finding is a reflection of the chemotactic effects under the action of mediators released at the lesion site that recruit large numbers of monocytes, which are activated (classical pathway) and differentiate into M1 macrophages^20^.

Our real-time PCR experiments and the MULTIPLEX/LUMINEX assays demonstrated increased levels of both mRNA (messenger ribonucleic acid) and the concentration of pro-inflammatory cytokines in liver tissue samples in the BD group. These findings are in agreement with the literature^19^. Indeed, during the inflammatory process, endothelial and liver cells express receptors for TNF- α and IL-1 β, leading to gene expression, with consequent protein synthesis of these cytokines^4^.

In parallel, circulating IL-6 is sequestered by the liver and functions as a trigger in the production and release of acute-phase proteins, such as C-reactive protein, serum amyloid A, α1-antichymotrypsin and fibrinogen^21^. Concomitantly, IL-6 acts on hepatocytes inducing the formation of CCL2/MCP-1, potentiating the local inflammatory infiltrate^22^.

Interestingly, the regulation of cytokine synthesis mainly involves mechanisms of a molecular nature (gene transcription) in which the mRNA gives the system an efficient control in the regulation of these factors^22,23^. Taken together, these observations allow us to conclude that the pathophysiology of BD is a dynamic process not restricted to the CNS, but with remarkable systemic effects, especially in vascular and hepatic tissues.

Despite reports in the literature regarding BD has broadened during the past few years, the precise mechanisms leading to immunologic response and augmented immunogenicity of organs after BD remain obscure. Thus, considered the gold standard of our work, the study of NF-κB expression and MHC molecules, evaluated by Gel-Shift assays and mRNA levels, showed increased expressions of both NF-κB and MHC class I and class II molecules in the liver, reinforcing the hypothesis that the immunogenicity of the graft may be orchestrated, to some extent, by NF-κB during an immune response^23^. Also, these data provide key insights for future therapies directed towards ameliorate the graft immunogenicity, and beneficial effect in outcome after transplantation.

The explanation for such findings may be closely anchored in the pathophysiological concepts of NF-kB. Therefore, NF-κB activation, via the canonical pathway, is triggered by signals that activate the IKK complex, with subsequent IκB phosphorylation (ubiquitination and degradation by proteasomes) in the cytoplasmic compartment, allowing the active dimeric form of NF-κB ( p65/p50 complex), translocate to the nucleus and bind to DNA regulating transcription of target genes^23,24^. It is currently known that NF-kB can be activated by TNF- α (TNFR receptors) and IL-1 β (IL-1R receptors), as well as by reactive oxygen species, adhesion molecules, in addition to the activation of TLRs^23^. Consequently, there is increased expression of class I MHC, in addition to adhesion molecules ICAM-1 and VCAM-1, all of which are essential in the post-transplant allorecognition mechanism. Other studies have also reported that NF-κB acts as a critical modulator in the control of apoptosis^24^. Additionally, other study showed that the major complex of the NFκB activation linked to inflammation response is linked to p50 p65^25^, in the present study, supershift assay suggest that the complex involved in BD is linked to the homodimer p65p65, a subunit with has transcriptional activity^26^. However, it cannot be ruled out that other subunits, such as p50, can also be involved in the NFκB activation since we do not have enough sample to run a supershift assay with the increasing concentration of antibodies against subunits p50 (1:20 and 1:10 dilution), p65 (1:40 and 1:20 dilution), c-Rel and p52 (1:10 dilution).

Following this conceptual line, some studies have shown that TNF- α potentiates class II MHC expression^27,28^ which leads to the conclusion that the balance between the coordinated actions between NF-κB and cytokines allows the regulation of immunogenicity in a process of positive feedback.

In addition, conducting research and developing new drugs are processes that are long and highly expensive, and it is not reasonable to deprive existing drugs, testing and maximizing their benefits with proven safety and adequately supported by science.

Finally, it is undoubted that the progressive understanding of immunopathogenesis during BD and the study of new therapies will continue to draw scientific progress in this area, which can translate into a great impact on the process of organ donation and transplantation.

## Conclusion

The current study found that BD is a multifaceted process that elicits both a systemic immune response and a local inflammatory response in liver tissue. Our findings strongly suggested that the immunogenicity of plasma and liver increased with time following brain death. In the domains of neurosurgery and clinical neurology, a detailed understanding based on immunologic response with regard to brain death is considered to be advantageous. These findings, taken combined, shed light on the immunological response in brain-dead organ donors. Future research should focus on gaining a better knowledge of the pathophysiology of BD in order to identify new and effective therapy targets.

## Supplementary Information

Supplementary Information.

## Data Availability

The datasets generated and analysed during the current study are not publicly available due to the database platform determines that experiments with less than 20 genes will not be accepted. Data are, however, available from the corresponding author upon reasonable request.
