# Supplementary material for: Immunomodulatory response in an experimental model of brain death
Source: Sci Rep. 2023 Jun 29;13:10524. doi: 10.1038/s41598-023-36629-9 (PMC10310852; doi:10.1038/s41598-023-36629-9)
Supplement: Supplementary file 1 — Supplementary Information. [file 41598_2023_36629_MOESM1_ESM.docx]

**Supplementary file S1**

Primers sequences used for Real Time PCR assays.

| **Gene** | **Sense and Antisense (5’ – 3’)** | **Produt**  **(pb)** |
| --- | --- | --- |
| **TNF-α** | 5’ TGGCCCAGACCCTCACACTCA 3’ | 541 |
|  | 5’ GGCTCAGCCACTCCAGCTGC 3’ |  |
| **IL-1β** | 5’ CCTTGTGCAAGTGTCTGAAGCAGC 3’ | 248 |
|  | 5’ GCCACAGCTTCTCCACAGCCA 3’ |  |
| **IL-6** | 5’ CCGGAGAGGAGACTTCACAGAGGA 3’ | 71 |
|  | 5’ AGCCTCCGACTTGTGAAGTGGTATA 3’ |  |
| **IL-10** | 5’ TCAGTCACATTTGTTTTCTGCAAA 3’ | 65 |
|  | 5’ CTGCAAAAGTGGAGCAGTCATT 3’ |  |
| **MHC**  **Class I** | 5’ TTCCTGCTACCGTTCCTCAC 3’ | 65 |
|  | 5’ GGTGTGAGTCCACATACCCA 3’ |  |
| **MHC Class II** | 5’ TCAGTCACATTTGTTTTCTGCAAA 3’ | 65 |
|  | 5’ CTGCAAAAGTGGAGCAGTCATT 3’ |  |
| **NF-κB** | 5’ ATCAAAGAGCTGGTGGAGGC 3’ | 188 |
|  | 5’ GAAGGCTGCCTGGATCACTT 3’ |  |
| **β-actin** | 5’ AGGAGTACGATGAGTCCGGCCC 3’ | 70 |
|  | 5’ GCAGCTCAGTAACAGTCCGCCT 3’ |  |

**Supplementary file S2**

Cycle Treshold used for Real Time PCR assays.

|  | **CYCLE TRESHOLD (CT)** | | | | | | |
| --- | --- | --- | --- | --- | --- | --- | --- |
| **Samples** | **β-actin** | **TNF-α** | **IL1-β** | **IL-6** | **IL-10** | **MHC-1** | **MHC-2** |
| **C1** | 18,08 | 23,48 | 22,94 | 24,86 | 29,15 | 28,33 | 26,50 |
| **C2** | 17,43 | 23,06 | 21,64 | 23,92 | 27,70 | 27,57 | 25,96 |
| **C3** | 17,43 | 23,51 | 22,95 | 24,02 | 30,16 | 28,89 | 27,00 |
| **C4** | 17,81 | 24,20 | 23,15 | 25,87 | 28,42 | 28,27 | 26,48 |
| **C5** | 17,94 | 23,72 | 22,08 | 24,57 | 28,22 | 27,78 | 28,10 |
| **C6** | 17,88 | 23,62 | 22,83 | 23,99 | 29,40 | 28,45 | 27,20 |
| **C7** | 17,85 | 23,32 | 22,45 | 24,86 | 28,78 | 29,01 | 26,33 |
| **C8** | 17,72 | 23,45 | 22,79 | 23,97 | 27,01 | 28,76 | 25,90 |
| **BD1** | 17,54 | 21,90 | 21,20 | 23,61 | 29,86 | 25,94 | 25,55 |
| **BD2** | 17,67 | 21,97 | 21,69 | 23,74 | 28,46 | 27,31 | 25,91 |
| **BD3** | 17,76 | 22,71 | 20,89 | 24,59 | 28,10 | 26,51 | 25,10 |
| **BD4** | 18,14 | 22,96 | 21,09 | 22,53 | 29,30 | 27,50 | 23,90 |
| **BD5** | 17,83 | 22,53 | 20,59 | 20,10 | 30,40 | 28,13 | 25,40 |
| **BD6** | 17,45 | 22,48 | 20,38 | 23,68 | 29,18 | 27,22 | 24,80 |
| **BD7** | 17,77 | 22,19 | 21,12 | 22,99 | 26,89 | 27,92 | 25,64 |
| **BD8** | 17,35 | 22,35 | 20,97 | 22,87 | 27,69 | 28,12 | 27,4 |

C = Control; BD = Brain Death
